# 5,7-Dibromo-2-methyl­quinolin-8-ol

**DOI:** 10.1107/S1600536811007434

**Published:** 2011-03-05

**Authors:** Nicole Schmidt, Anke Schwarzer, Edwin Weber

**Affiliations:** a Institut für Organische Chemie, TU Bergakademie Freiberg, Leipziger Strasse 29, D-09596 Freiberg/Sachsen, Germany

## Abstract

In the title compound, C_10_H_7_Br_2_NO, the mol­ecule possesses a planar geometry with an r.m.s deviation of 0.0383 Å for all non-H atoms. The crystal structure displays O—H⋯N and C—H⋯O hydrogen bonding, as well as Br⋯Br contacts [3.6284 (4) Å].

## Related literature

For a review of hy­droxy­quinolines in supra­molecular chemistry, see: Albrecht *et al.* (2008 ▶). Bei *et al.* (1997 ▶) report on group 4 metal alkyl complexes. The crystal structure of the parent 8-hy­droxy­quinoline is described by Banerjee & Saha (1986 ▶) and Roychowdhury *et al.* (1978 ▶). Choi & Chi (2004 ▶) used the title compound as the starting material for alkyl­amino-substituted quinoline-5,8-diones. For halogen inter­actions in mol­ecular crystal structures, see: Awwadi *et al.* (2006 ▶); Brammer *et al.* (2001 ▶); Metrangolo *et al.* (2008 ▶).
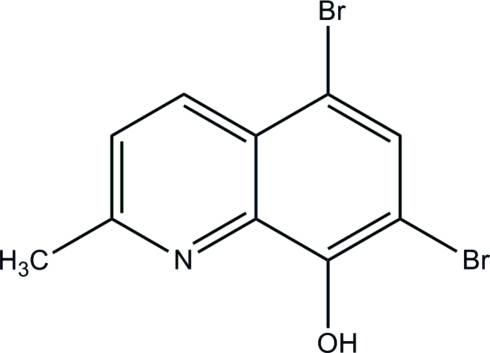


## Experimental

### 

#### Crystal data

C_10_H_7_Br_2_NO
*M* *_r_* = 316.99
Monoclinic, 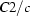

*a* = 22.2221 (5) Å
*b* = 4.0479 (1) Å
*c* = 21.7221 (4) Å
β = 102.167 (1)°
*V* = 1910.07 (7) Å^3^
*Z* = 8
Mo *K*α radiation
μ = 8.45 mm^−1^
*T* = 93 K
0.40 × 0.24 × 0.22 mm

#### Data collection

Bruker SMART CCD area-detector diffractometer
Absorption correction: multi-scan (*SADABS*; Bruker, 2007 ▶) *T*_min_ = 0.133, *T*_max_ = 0.258
13437 measured reflections
1727 independent reflections
1629 reflections with *I* > 2σ(*I*)
*R*_int_ = 0.025

#### Refinement

*R*[*F* ^2^ > 2σ(*F* ^2^)] = 0.017
*wR*(*F* ^2^) = 0.046
*S* = 1.11
1727 reflections
129 parameters
H-atom parameters constrained
Δρ_max_ = 0.36 e Å^−3^
Δρ_min_ = −0.60 e Å^−3^

### 

Data collection: *SMART* (Bruker, 2007 ▶); cell refinement: *SAINT* (Bruker, 2007 ▶); data reduction: *SAINT*; program(s) used to solve structure: *SHELXS97* (Sheldrick, 2008 ▶); program(s) used to refine structure: *SHELXL97* (Sheldrick, 2008 ▶); molecular graphics: *SHELXTL* (Sheldrick, 2008 ▶); software used to prepare material for publication: *SHELXTL*.

## Supplementary Material

Crystal structure: contains datablocks global, I. DOI: 10.1107/S1600536811007434/im2267sup1.cif

Structure factors: contains datablocks I. DOI: 10.1107/S1600536811007434/im2267Isup2.hkl

Additional supplementary materials:  crystallographic information; 3D view; checkCIF report

## Figures and Tables

**Table 1:** Hydrogen-bond geometry (Å, °)

*D*—H⋯*A*	*D*—H	H⋯*A*	*D*⋯*A*	*D*—H⋯*A*
O1—H1⋯N1^i^	0.84	1.92	2.707 (2)	157
C10—H10*A*⋯O1^ii^	0.98	2.52	3.342 (3)	141

Symmetry codes: (i) 
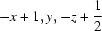
; (ii) 
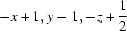
.

**Fig. 1.**
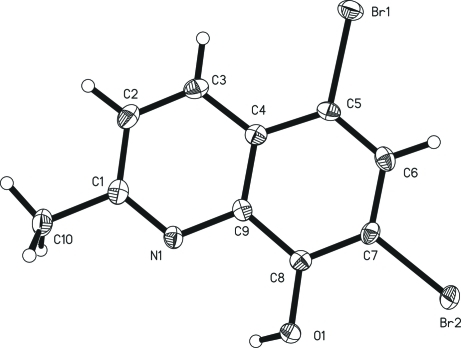
Perspective view of (I), showing 50% probability displacementellipsoids for the non-H atoms.

**Fig. 2.**
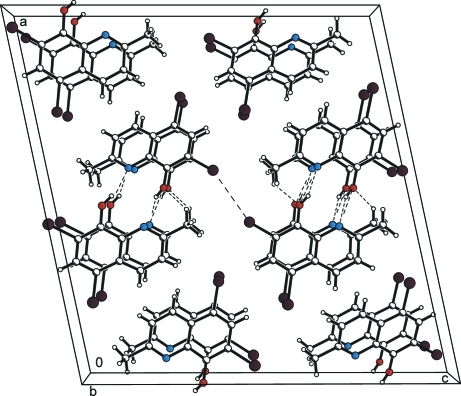
View of the crystal packing of the title compound along the crystallographic b axis. C–H···O, O–H···N and Br···Br contacts are shown as broken lines.

## supplementary crystallographic information

### Comment

The molecular shape of the title compound is best described by the planarity of the molecule (Fig. 1), expressed by the RMS deviation of all non-hydrogen fitted atoms being 0.0383 Å. Molecular dimers are formed by a conventional hydrogen bridge, O1—H1···N1 [*d* = 2.707 (2) Å, *θ* = 157°] (Fig. 2) that is also found in the structure of the parent 8-hydroxyquinoline (Banerjee & Saha, 1986). In addition, a C—H···O contact creates chains along the crystallographic *b* axis. Distances between adjacent aromatic planes of 4.1 Å indicate the absence of π stacking interactions. However, halogen interactions of type I mode (Awwadi *et al.* 2006) represented by the Br2···Br2 contact [*d* = 3.6284 (4) Å, *θ*_1_ = *θ*_2_ = 143.3°] connect the formed dimers. Considering analogous dimer formation in the parent 8-hydroxyquinoline, this particular halogen contact is largely attributable to crystal packing effects.

### Experimental

As described by Choi & Chi (2004), 5 ml of bromine in MeOH (50 ml) was added to a mixture of 8-hydroxy-2-methylquinoline (5.0 g, 31.4 mmol), NaHCO_3_ (5 g) and MeOH (50 ml). After stirring for 5 min at room temperature, Na_2_SO_3_ (2.5 g) was added, and then the mixture was filtered and washed with H_2_O (100 ml). The white solid was dried *in vacuo* to give the title compound as raw product (8.9 g, 89%). Recrystallization from boiling and slowly cooling to room temperature ethanol yielded single crystals suitable for X-ray crystallography.

### Refinement

H atoms were positioned geometrically and allowed to ride on their parent atoms, with O—H = 0.84 Å, C—H = 0.95–0.98 Å and *U*_iso_(H) = 1.2–1.5 *U*_eq_(parent atom).

### Crystal data

**Table:**

C_10_H_7_Br_2_NO	*F*(000) = 1216
*M**_r_* = 316.99	*D*_x_ = 2.205 Mg m^−^^3^
Monoclinic, *C*2/*c*	Mo *K*α radiation, λ = 0.71073 Å
Hall symbol: -C 2yc	Cell parameters from 9610 reflections
*a* = 22.2221 (5) Å	θ = 2.4–29.2°
*b* = 4.0479 (1) Å	µ = 8.45 mm^−^^1^
*c* = 21.7221 (4) Å	*T* = 93 K
β = 102.167 (1)°	Piece, colourless
*V* = 1910.07 (7) Å^3^	0.40 × 0.24 × 0.22 mm
*Z* = 8	

### Data collection

**Table:**

Bruker SMART CCD area-detector diffractometer	1727 independent reflections
Radiation source: fine-focus sealed tube	1629 reflections with *I* > 2σ(*I*)
graphite	*R*_int_ = 0.025
φ and ω scans	θ_max_ = 25.2°, θ_min_ = 1.9°
Absorption correction: multi-scan (*SADABS*; Bruker, 2007)	*h* = −26→25
*T*_min_ = 0.133, *T*_max_ = 0.258	*k* = −4→4
13437 measured reflections	*l* = −26→26

### Refinement

**Table:**

Refinement on *F*^2^	Primary atom site location: structure-invariant direct methods
Least-squares matrix: full	Secondary atom site location: difference Fourier map
*R*[*F*^2^ > 2σ(*F*^2^)] = 0.017	Hydrogen site location: inferred from neighbouring sites
*wR*(*F*^2^) = 0.046	H-atom parameters constrained
*S* = 1.11	*w* = 1/[σ^2^(*F*_o_^2^) + (0.0262*P*)^2^ + 2.9807*P*] where *P* = (*F*_o_^2^ + 2*F*_c_^2^)/3
1727 reflections	(Δ/σ)_max_ = 0.001
129 parameters	Δρ_max_ = 0.36 e Å^−^^3^
0 restraints	Δρ_min_ = −0.60 e Å^−^^3^

### Special details

**Table:**

Geometry. All e.s.d.'s (except the e.s.d. in the dihedral angle between two l.s. planes) are estimated using the full covariance matrix. The cell e.s.d.'s are taken into account individually in the estimation of e.s.d.'s in distances, angles and torsion angles; correlations between e.s.d.'s in cell parameters are only used when they are defined by crystal symmetry. An approximate (isotropic) treatment of cell e.s.d.'s is used for estimating e.s.d.'s involving l.s. planes.
Refinement. Refinement of *F*^2^ against ALL reflections. The weighted *R*-factor *wR* and goodness of fit *S* are based on *F*^2^, conventional *R*-factors *R* are based on *F*, with *F* set to zero for negative *F*^2^. The threshold expression of *F*^2^ > σ(*F*^2^) is used only for calculating *R*-factors(gt) *etc*. and is not relevant to the choice of reflections for refinement. *R*-factors based on *F*^2^ are statistically about twice as large as those based on *F*, and *R*- factors based on ALL data will be even larger.

### Fractional atomic coordinates and isotropic or equivalent isotropic displacement parameters (Å^2^)

**Table:**

	*x*	*y*	*z*	*U*_iso_*/*U*_eq_	
Br1	0.226304 (10)	0.34054 (6)	0.083369 (10)	0.02104 (8)	
Br2	0.431764 (10)	1.01225 (5)	0.033388 (9)	0.02065 (8)	
N1	0.42288 (8)	0.6326 (4)	0.26148 (8)	0.0151 (4)	
O1	0.47717 (7)	0.9601 (4)	0.17443 (7)	0.0197 (3)	
H1	0.5012	0.8441	0.2009	0.030*	
C1	0.39566 (10)	0.4874 (5)	0.30307 (10)	0.0168 (4)	
C2	0.33816 (10)	0.3287 (5)	0.28492 (10)	0.0198 (5)	
H2	0.3204	0.2213	0.3157	0.024*	
C3	0.30807 (10)	0.3291 (5)	0.22338 (10)	0.0186 (5)	
H3	0.2693	0.2223	0.2111	0.022*	
C4	0.33477 (10)	0.4888 (5)	0.17805 (10)	0.0144 (4)	
C5	0.30664 (10)	0.5140 (5)	0.11344 (10)	0.0155 (4)	
C6	0.33502 (10)	0.6709 (5)	0.07179 (10)	0.0174 (4)	
H6	0.3154	0.6859	0.0286	0.021*	
C7	0.39324 (10)	0.8094 (5)	0.09334 (10)	0.0150 (4)	
C8	0.42345 (10)	0.8007 (5)	0.15583 (10)	0.0145 (4)	
C9	0.39347 (10)	0.6360 (5)	0.19921 (9)	0.0137 (4)	
C10	0.42720 (11)	0.4996 (6)	0.37143 (10)	0.0224 (5)	
H10A	0.4668	0.3840	0.3774	0.034*	
H10B	0.4012	0.3923	0.3968	0.034*	
H10C	0.4342	0.7303	0.3847	0.034*	

### Atomic displacement parameters (Å^2^)

**Table:**

	*U*^11^	*U*^22^	*U*^33^	*U*^12^	*U*^13^	*U*^23^
Br1	0.01485 (13)	0.02683 (14)	0.02053 (13)	−0.00403 (8)	0.00170 (9)	−0.00389 (8)
Br2	0.02015 (14)	0.02857 (14)	0.01417 (13)	−0.00252 (9)	0.00577 (9)	0.00366 (8)
N1	0.0131 (9)	0.0189 (9)	0.0133 (8)	0.0042 (7)	0.0024 (7)	0.0016 (7)
O1	0.0130 (8)	0.0279 (9)	0.0169 (8)	−0.0037 (6)	0.0001 (6)	0.0048 (6)
C1	0.0168 (11)	0.0181 (11)	0.0160 (10)	0.0068 (8)	0.0048 (9)	0.0025 (8)
C2	0.0196 (12)	0.0220 (12)	0.0198 (11)	0.0029 (9)	0.0088 (9)	0.0040 (9)
C3	0.0142 (11)	0.0193 (11)	0.0233 (11)	0.0004 (8)	0.0063 (9)	0.0011 (9)
C4	0.0124 (11)	0.0144 (10)	0.0172 (11)	0.0037 (8)	0.0045 (8)	−0.0007 (8)
C5	0.0114 (10)	0.0159 (10)	0.0187 (10)	0.0005 (8)	0.0021 (8)	−0.0027 (8)
C6	0.0174 (11)	0.0210 (11)	0.0133 (10)	0.0041 (9)	0.0022 (8)	−0.0015 (8)
C7	0.0157 (11)	0.0168 (10)	0.0143 (10)	0.0023 (8)	0.0071 (8)	0.0017 (8)
C8	0.0113 (10)	0.0153 (10)	0.0174 (10)	0.0032 (8)	0.0039 (8)	0.0003 (8)
C9	0.0131 (11)	0.0152 (10)	0.0130 (10)	0.0043 (8)	0.0030 (8)	−0.0011 (8)
C10	0.0236 (13)	0.0286 (13)	0.0152 (11)	0.0026 (9)	0.0047 (9)	0.0035 (9)

### Geometric parameters (Å, °)

**Table:**

Br1—C5	1.901 (2)	C3—H3	0.9500
Br2—C7	1.890 (2)	C4—C5	1.415 (3)
N1—C1	1.326 (3)	C4—C9	1.420 (3)
N1—C9	1.373 (3)	C5—C6	1.364 (3)
O1—C8	1.342 (3)	C6—C7	1.397 (3)
O1—H1	0.8400	C6—H6	0.9500
C1—C2	1.410 (3)	C7—C8	1.382 (3)
C1—C10	1.503 (3)	C8—C9	1.429 (3)
C2—C3	1.363 (3)	C10—H10A	0.9800
C2—H2	0.9500	C10—H10B	0.9800
C3—C4	1.409 (3)	C10—H10C	0.9800
			
C1—N1—C9	119.01 (18)	C5—C6—H6	120.3
C8—O1—H1	109.5	C7—C6—H6	120.3
N1—C1—C2	121.88 (19)	C8—C7—C6	122.87 (19)
N1—C1—C10	118.3 (2)	C8—C7—Br2	119.40 (16)
C2—C1—C10	119.8 (2)	C6—C7—Br2	117.73 (15)
C3—C2—C1	120.1 (2)	O1—C8—C7	120.03 (19)
C3—C2—H2	119.9	O1—C8—C9	122.33 (18)
C1—C2—H2	119.9	C7—C8—C9	117.52 (19)
C2—C3—C4	119.6 (2)	N1—C9—C4	121.94 (19)
C2—C3—H3	120.2	N1—C9—C8	117.59 (18)
C4—C3—H3	120.2	C4—C9—C8	120.45 (18)
C3—C4—C5	124.3 (2)	C1—C10—H10A	109.5
C3—C4—C9	117.38 (19)	C1—C10—H10B	109.5
C5—C4—C9	118.34 (19)	H10A—C10—H10B	109.5
C6—C5—C4	121.5 (2)	C1—C10—H10C	109.5
C6—C5—Br1	118.43 (16)	H10A—C10—H10C	109.5
C4—C5—Br1	120.05 (16)	H10B—C10—H10C	109.5
C5—C6—C7	119.32 (19)		
			
C9—N1—C1—C2	1.8 (3)	C6—C7—C8—O1	−174.87 (19)
C9—N1—C1—C10	−177.25 (18)	Br2—C7—C8—O1	5.8 (3)
N1—C1—C2—C3	−1.8 (3)	C6—C7—C8—C9	1.2 (3)
C10—C1—C2—C3	177.2 (2)	Br2—C7—C8—C9	−178.10 (15)
C1—C2—C3—C4	0.0 (3)	C1—N1—C9—C4	0.0 (3)
C2—C3—C4—C5	−177.7 (2)	C1—N1—C9—C8	178.67 (18)
C2—C3—C4—C9	1.6 (3)	C3—C4—C9—N1	−1.7 (3)
C3—C4—C5—C6	−179.7 (2)	C5—C4—C9—N1	177.67 (18)
C9—C4—C5—C6	1.0 (3)	C3—C4—C9—C8	179.69 (19)
C3—C4—C5—Br1	2.5 (3)	C5—C4—C9—C8	−1.0 (3)
C9—C4—C5—Br1	−176.86 (14)	O1—C8—C9—N1	−2.8 (3)
C4—C5—C6—C7	0.1 (3)	C7—C8—C9—N1	−178.78 (18)
Br1—C5—C6—C7	177.96 (15)	O1—C8—C9—C4	175.90 (18)
C5—C6—C7—C8	−1.3 (3)	C7—C8—C9—C4	−0.1 (3)
C5—C6—C7—Br2	178.09 (16)		

### Hydrogen-bond geometry (Å, °)

**Table:**

*D*—H···*A*	*D*—H	H···*A*	*D*···*A*	*D*—H···*A*
O1—H1···N1^i^	0.84	1.92	2.707 (2)	157
C10—H10A···O1^ii^	0.98	2.52	3.342 (3)	141

Symmetry codes: (i) −*x*+1, *y*, −*z*+1/2; (ii) −*x*+1, *y*−1, −*z*+1/2.
